# High prevalence of norovirus in children with sporadic acute gastroenteritis in Manaus, Amazon Region, northern Brazil

**DOI:** 10.1590/0074-02760160357

**Published:** 2017-06

**Authors:** Samya Thalita Picanço da Costa, Tulio Machado Fumian, Ian Carlos Gomes de Lima, Jones Anderson Monteiro Siqueira, Luciana Damascena da Silva, Juliana das Mercês Hernández, Maria Silvia Souza de Lucena, Tammy Kathlyn Amaral Reymão, Luana da Silva Soares, Joana D’Arc Pereira Mascarenhas, Yvone Benchimol Gabbay

**Affiliations:** 1 Universidade do Estado do Pará, Programa de Pós-Graduação em Biologia Parasitária na Amazônia, Belém, PA, Brasil; 2 Fundação Oswaldo Cruz-Fiocruz, Instituto Oswaldo Cruz, Laboratório de Virologia Comparada e Ambiental, Rio de Janeiro, RJ, Brasil; 3 Secretaria de Vigilância em Saúde, Instituto Evandro Chagas, Seção de Virologia, Ananindeua, PA, Brasil; 4 Instituto Evandro Chagas, Programa de Pós-Graduação em Virologia, Ananindeua, PA, Brasil

**Keywords:** norovirus, Amazon Region, children, diarrhoea

## Abstract

**BACKGROUND:**

Norovirus (NoV) is a major cause of acute gastroenteritis (AGE) worldwide, especially in children under five years. Studies involving the detection and molecular characterisation of NoV have been performed in Brazil, demonstrating its importance as an etiological agent of AGE.

**OBJECTIVES:**

The objectives of this study were to investigate the frequency of human NoV and to genotype the strains isolated from 0-14-year-old patients of AGE in Manaus, Brazil, over a period of two years.

**METHODS:**

A total of 426 faecal samples were collected between January 2010 and December 2011. All samples were tested for the presence of NoV antigens using a commercial enzyme immunoassay kit. RNA was extracted from all faecal suspensions and reverse transcription-polymerase chain reaction (RT-PCR) for the NoV-polymerase partial region was performed as a trial test. Positive samples were then subjected to PCR with specific primers for partial capsid genes, which were then sequenced.

**FINDINGS:**

NoV was detected in 150 (35.2%) faecal samples, for at least one of the two techniques used. NoV was detected in children from all age groups, with the highest positivity observed among the group of 1-2 years old. Clinically, fever was verified in 43% of the positive cases and 46.3% of the negative cases, and vomiting was observed in 75.8% and 70.8% cases in these groups, respectively. Monthly distribution showed that the highest positivity was observed in January 2010 (81.2%), followed by February and April 2010 and March 2011, when the positivity rate reached almost 50%. Phylogenetic analyses performed with 65 positive strains demonstrated that 58 (89.2%) cases of NoV belonged to genotype GII.4, five (7.7%) to GII.6, and one (1.5%) each to GII.7 and GII.3.

**MAIN CONCLUSIONS:**

This research revealed a high circulation of NoV GII.4 in Manaus and contributed to the understanding of the importance of this virus in the aetiology of AGE cases, especially in a region with such few studies available.

Noroviruses (NoV) are key pathogens causing nonbacterial acute gastroenteritis (AGE) in all age groups, and are responsible for almost 50% of AGE outbreaks worldwide. The AGE caused by NoV is usually expressed as diarrhoea, vomiting, nausea, mild fever, and abdominal pain, which can eventually lead to dehydration and death, mainly in young children and the elderly. Outbreaks of AGE associated with NoV have been reported in semi-closed environments such as hospitals, aged care homes, cruise ships and prisons (Green 2013).

NoV are small, rounded, non-enveloped viruses with a single-stranded, positive-sense, polyadenylated RNA genome of about 7,500 nucleotides (nt) in length. The genome contains three open reading frames (ORFs), denominated ORF 1, 2, and 3. NoV have been classified into six genogroups (GI to GVI) based on their VP1 amino acid sequence (Ramani et al. 2014); only GI, GII and GIV have been associated with human infection (Green 2013). These genogroups are further divided into at least 36 genotypes (Kroneman et al. 2013), but studies have demonstrated a predominance of the GII genotype, specifically GII.4 (Zeng et al. 2012).

NoV-GII.4 is genetically highly heterogeneous, and new strains frequently arise because of antigenic drift in VP1 and genetic recombination between pre-existing NoV strains. Approximately every two or three years, global epidemics of AGE have been related to this genotype. Some of these variants, such as Farmington Hills_2002, Hunter_2004, Yerseke_2006a, Den Haag_2006b, Apeldoorn_2008, New Orleans_2009, and the most recently reported Sydney_2012, have a global distribution, and are the major variants responsible for NoV AGE outbreaks worldwide (Van Beek et al. 2013, Ramani et al. 2014).

In Brazil, several studies involving the detection and molecular characterisation of NoV from sporadic AGE cases, outbreaks, and hospitalisation from different places have been performed, demonstrating the importance of this virus as an aetiological agent of AGE (Castilho et al. 2006, Barreira et al. 2010, Fioretti et al. 2011, da Silva et al. 2013, Siqueira et al. 2013).

Regarding Manaus city, the current knowledge about NoV is limited to two environmental studies carried out within the city using river water samples (Miagostovich et al. 2008, Vieira et al. 2016), and to one showing unusual recombination types of NoV in sporadic cases of diarrhoea observed in patients who visited public health facilities (Hernández et al. 2016). Manaus is the capital city of Amazonas state, located in northern Brazil, and with a population of more than 2 million, it is considered the most populous city of the Amazon Region. Manaus is located in the middle of the Amazon rainforest, and access to the city is primarily by boat or airplane. As of yet, there is no clinical study describing the NoV epidemiological profile in this city. Therefore, our aim was to investigate the occurrence of NoV infections in samples collected from children with AGE in Manaus, between January 2010 and December 2011. We also analysed the genetic diversity of NoV according to genogroup and genotypes.

## MATERIALS AND METHODS

*Stool samples* - A total of 426 faecal specimens were collected in Manaus, Amazonas (162 samples in 2010 and 264 in 2011). Samples from children hospitalised with AGE (0-14 years old) were collected by the Central Laboratory of Amazonas state (LACEN-AM) and then forwarded to the Evandro Chagas Institute (IEC) by the Brazilian Network Surveillance Program of Viral Gastroenteritis.

*Immunoenzymatic assay* - Samples were initially tested for the presence of NoV antigens using a third generation commercial Ridascreen^®^ Norovirus EIA kit (Darmstadt, Germany), according to the manufacturer’s instructions. This test is a qualitative solid phase that utilises a combination of NoV GI- and GII-specific monoclonal antibodies.

*RNA extraction and cDNA synthesis* - Viral RNA was extracted from 300 µL of a 10% (w/v) faecal suspension in Tris-HCl-Ca^2+^ buffer, pH 7.2, by the guanidine isothiocyanate/silica method, as described previously (Boom et al. 1990). The synthesis of complementary DNA was carried out using a random primer, Pd(N)_6_ (Invitrogen^®^, Eugene, Oregon, USA) and the Superscript™ II RNase H Reverse Transcriptase (Invitrogen^®^, Eugene, Oregon, USA).

*Molecular detection* - Polymerase chain reaction (PCR) amplification was performed in all samples using a pool of primers for the B region (nt: 5093-5305; 213 bp) of the NoV polymerase gene (ORF1): Mon 431/433 and 432/434 to detect GI and GII, respectively (Anderson et al. 2001).

*Molecular characterisation* - NoV-positive samples were subjected to another round of PCR using a set of primers targeting region D, a partial region located in the capsid (ORF-2) (Vinjé et al. 2004). First, PCR was performed for GII using a set of primers including Cap C, D1, and D3 (nt: 6432-6684; 253 bp), and then negative samples were tested using the primers Cap A, B1, and B2 (nt: 6738-6914; 177 bp), specific to GI. The amplicons obtained were purified with the commercial kit QIAquick PCR Purification Kit (Qiagen^®^, Valencia, CA, USA) and quantified using the Low DNA Mass Ladder (Invitrogen^®,^ Carlsbad, CA, USA).

DNA sequencing was performed using an ABI Prism BigDye Terminator Cycle Sequencing Ready Reaction Kit and the ABI Prism 3130xl DNA Sequencer (Applied Biosystems^®^, Foster City, CA, USA). The sequences were edited using the BioEdit Sequence Alignment Editor (v.7.0.9.1) software. Consensual sequences were used to construct a phylogenetic dendrogram by the neighbour-joining method, using a matrix of genetic distances established under the Kimura two-parameter model with 2000 bootstrap replications for branch support using MEGA 6 (Tamura et al. 2011). Sequences identified in this study were submitted to the GenBank database [National Center for Biotechnology Information, US (www.ncbi.nlm.nih.gov)] under accession numbers: KX232361-KX232423.

*Statistical analyses* - Statistical analyses were performed using BioEstat 5.0 (Ayres et al. 2007). The screening test was performed to compare the sensitivity and speciﬁcity of the results obtained by EIA in comparison with those from reverse transcription-PCR (RT-PCR), which is considered the gold standard. The Kappa test was used to evaluate the reproducibility of the EIA, and the odds ratio (OR) test was used to analyse the differences in NoV infection rates among the various age groups. A simple linear regression was used to assess the relationship between the positive cases with the monthly rainfall parameters, as well as the presence of vomiting and fever. P-values ≤ 0.05 were considered to be statistically signiﬁcant.

*Ethics statement* - The study protocol and consent forms were approved by the Human Research Ethics Committee of the Evandro Chagas Institute (CEP/IEC 038/2011).

## RESULTS

An overall positivity of 35.2% (150/426) was observed, with 104 positive samples detected using a combination of both methods, 23 using RT-PCR only, and 23 using EIA only. A higher percentage of positivity was obtained in 2010 (40.7%) than that observed in 2011 (31.8%). We used the screening test [95% confidence interval (CI)] to verify the sensitivity/specificity of the EIA method in comparison with RT-PCR and obtained values of 81.9% and 92.3%, respectively. In order to verify the reproducibility of the technique, we used the Kappa test that showed statistical significance (Kappa = 0.7420; p < 0.0001) with good replicability and a concordance rate of 89.2% between both methods.

**Fig. 1:**
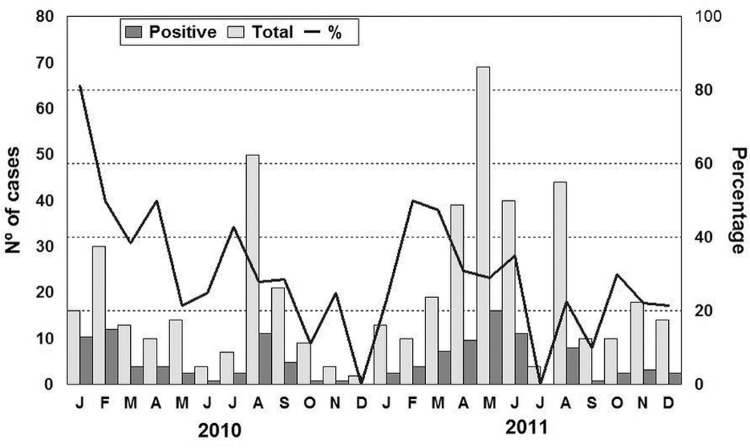
: monthly distribution of norovirus infection in 426 faecal specimens collected from children with sporadic acute gastroenteritis in Manaus, Brazil, between January 2010 and December 2011.

We detected NoV throughout the year, with a higher positivity rate in January 2010 (81.2%), followed by February and April 2010 and March 2011, when the positivity rates reached almost 50% (Fig. 1). The NoV was detected in stool samples of children from all age groups, but the highest positivity was observed among the group of 1 to 2-year-olds (Table). Children up to two years of age showed statistical significance (p = 0.03) when compared with the oldest group.

**TABLE:** Distribution of norovirus-positive cases for age group, among children with acute gastroenteritis in Manaus, Amazonas, from January 2010 to December 2011

Age group (months/years)	Positive samples/total of cases (%)
0-6m^*^	16/46 (34.8%)
> 6-12m^*^	57/157 (36.3%)
> 1-2y^*^	57/134 (42.5%)
> 2-5y	14/65 (21.5%)
> 5-14y	6/24 (25%)

Total	150/426 (35.2%)

*: children until two years; odds ratio = 1.88 (95% confidence interval = 1.08-3.27); p = 0.03; number needed to cause one adverse event at time *t* (NNH) = 8.

Analysis of the records containing clinical information demonstrated that fever was observed in 43% of positive cases (46/107) and 46.3% (82/177) of negative cases. Vomiting was verified in 75.8% (91/120) and 70.8% (148/209) of positive and negative cases, respectively. However, NoV was not associated with fever (p = 0.6129) or vomiting (p = 0.2919).

**Fig. 2:**
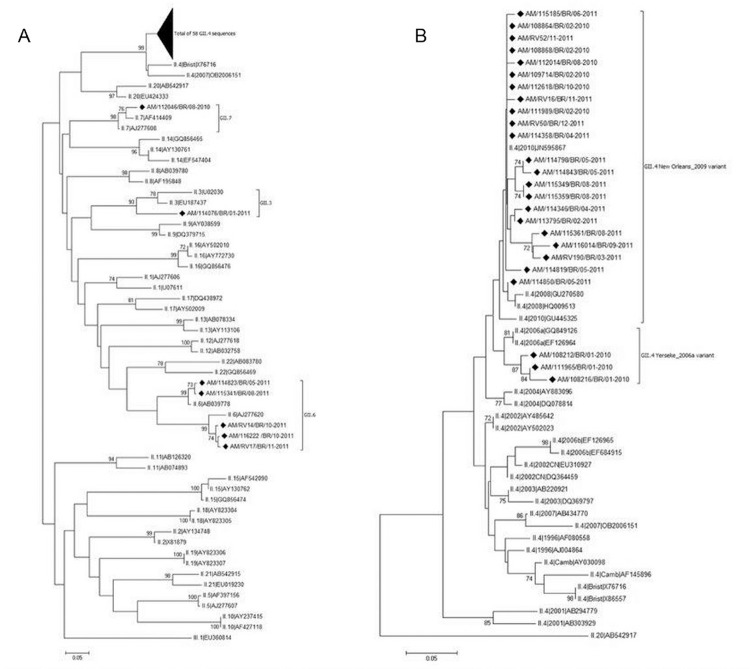
: phylogenetic analyses of norovirus GII sequences obtained from children with diarrhoea from Manaus, Brazil, between January 2010 and December 2011, based on a 253 bp region within the capsid. (A) Phylogenetic tree of norovirus GII genotypes; (B) phylogenetic tree of GII.4 variants. References strains of NoV genotypes are named according to GenBank with their respective accession numbers. Brazilian strains are marked with a filled diamond. The scale bar at the bottom of the tree indicates distance. Bootstrap values (2000 replicates) are shown at the branch nodes and values lower than 60% are not shown. The codes representing the positive samples are in bold and are organised as follows: study area (Amazonas)/sample code/country of collection (Brazil)/month-year of collection.

Of the 127 samples with a NoV-positive result by RT-PCR (polymerase region), 51.2% (65/127) were sequenced using primers specific for a segment of the capsid region (region D). Phylogenetic analysis demonstrated that 58 (89.2%) belonged to the genotype GII.4, five (7.7%) to GII.6, and one (1.5%) each to GII.7 and GII.3 (Fig. 2). Among the 58 GII.4 strains, we detected the circulation of two different variants: three strains (5.2%) were classified as the variant Yerseke_2006a in January 2010, and the remaining strains were classified as the variant New Orleans_2009 in 2010 and 2011 (Fig. 2).

## DISCUSSION

The combination of the two methods (EIA and RT-PCR) in the diagnosis of NoV provided a high percentage of detection (35.2%), which was similar or superior to those obtained in some previous studies conducted in Brazil, including those in Belém and Rio Branco, both in the Amazon Region, northern Brazil (da Silva et al. 2013, Siqueira et al. 2013). Studies performed around the world, using PCR targeting to the same region B, showed similar positivity percentages as the results of this study, ranging up to 33.3% (Castilho et al. 2006).

The NoV was observed to be circulating in patients throughout the whole period of the study. Among the patients, the highest prevalence was observed in children under two years of age (38.6%, 130/337). These results differ from the findings by Aragão et al. (2010), which showed a lower prevalence of NoV (13.2%, 34/257) in the same age group. Statistical analyses supported the finding that children from Manaus under two years of age were eight times more likely to develop AGE caused by NoV than those of other ages [number needed to cause one adverse event at time *t* (NNH) = 8], considering a period of two years. Similar results were observed by Siqueira et al. (2013) in children hospitalised in Belém, also in northern Brazil, where the age group most affected by NoV comprised children up to two years old; however, this group had a lower NNH (n = 3), showing a higher attack rate, with three times the likelihood of developing NoV disease. NoV had a predominant role in cases of AGE in recent years among people of all ages, but children under two years old are still the most vulnerable portion of the population (Patel et al. 2008).

Due to the high genetic variability of NoV, molecular characterisation of the virus to distinguish the different genotypes is important. The analysis of the VP1 protein fragment of the capsid is considered efficient for carrying out the genotyping of NoV, as previously described (Vinjé et al. 2004). In this study, the genotype GII.4 was the most prevalent, observed in 89.2% (58/65) of samples genotyped, present in almost every month over the two years. These results corroborate with those of other studies conducted in Brazil (Barreira et al. 2010, Siqueira et al. 2013) and in other countries (Park et al. 2010, Vega et al. 2011, Gómez-Santiago et al. 2012), confirming the high circulation and impact of this genotype in cases of AGE.

The NoV-GII.4 variant Yerseke_2006a was detected (5.2%) only in January 2010. Some studies have reported its circulation by mid-2006, being later replaced by the variant 2006b (Tu et al. 2008). Likewise, it was detected in several countries such as Brazil, China, and Belgium (Fioretti et al. 2011, Mathijs et al. 2011, Zeng et al. 2012). New Orleans_2009 was the most prevalent (93.1%) circulating variant in both years. This result is in agreement with the epidemiological pattern found worldwide after the year 2009 (Vega et al. 2011). The first report of this variant occurred in the United States in the winter of 2009/2010, being involved with a large number of outbreaks (Yen et al. 2011). Among strains genotyped as Yerseke_2006a and New Orleans_2009, the nucleotide identity ranged from 98.1% to 98.6%, and 94.4% to 100%, respectively. Between both variant strains, the nucleotide identity ranged from 91.1% to 94.8%.

Genotypes GII.6, GII.3, and GII.7 were observed at low frequencies (7.7%, 1.5%, and 1.5%, respectively). The genotype GII.6 was the second most prevalent in this study, in agreement with previous reports (Ferreira et al. 2010, Andrade et al. 2014). Some studies have demonstrated that the circulation of this genotype is common (Zeng et al. 2012, Huang et al. 2013), which highlights the need for the surveillance of this strain in cases of AGE caused by NoV. The other genotypes found in this study (GII.3 and GII.7) have also been previously described at a low frequency in Pará (Aragão et al. 2010), São Paulo (Castilho et al. 2006), and Rio de Janeiro (Fioretti et al. 2011). Moreover, another survey that was conducted in China, involving children with diarrhoea, revealed a much higher percentage (23.8%) of genotype GII.3 (Zeng et al. 2012).

Some limitations of this study include the lack of full information in the clinical records received, and sometimes the amount of material received was insufficient for conducting all of the tests. These aspects hindered a more detailed analysis of the childrens’ epidemiological profiles. An additional limitation was that since only NoV GII-negative samples were tested for the capsid region with GI specificity, co-infections of GI and GII were not investigated. However, the combined use of EIA and RT-PCR demonstrated that the association of two or more different techniques might improve the NoV-detection rates, as demonstrated in this study.

Several studies conducted in the last decade have led to considerable advances in the understanding of the epidemiology and diversity of NoV, emphasising the relevance of the surveillance networks to identify the circulating NoV genotypes. The development of NoV surveillance systems is important for understanding the circulation, evolutionary processes, the emergence, and the spread of NoV disease (Mans et al. 2016).

This study identified a high number of NoV-positive GII.4 strains in Manaus, Brazil, and contributes to an understanding of the role of NoV in AGE cases. The diagnosis of infection with these viruses, especially in sporadic cases of AGE, is extremely important in order to prevent outbreaks and to eliminate possible sources of contamination. For this reason, the use of rapid, specific, and sensitive detection methods it is essential. Moreover, the knowledge regarding the major NoV circulating genotypes can support the formulation of new efficacious treatments as well as future vaccines.
